# Hôpital St. Antoine

**Published:** 1830-10-01

**Authors:** 


### HOPITAL ST. ANTOINE.

#### I. CASES OF INTERNAL STRANGULATION OF INTESTINES, FOR WHICH AN OPERATION IS PROPOSED.*

*Case* 1. A young woman was admitted into the St. Antoine Hospital on the 28th February, 1830, with vomiting, pain in the epigastrium and right iliac region, diarrhœa, and symptoms of phthisis pulmonalis. Leeches were applied to the epigastrium and iliac region with relief, but on the night of the 3d, she was seized again with violent pain in the belly, vomiting, and no stools; the pulse was small, frequent, and regular. A bleeding procured alleviation, but the symptoms returned next day, and were met by active antiphlogistic measures. On the 5th, the pain continued, and the neighbourhood of the umbilicus was tympanitic; incessant vomiting was experienced during the night; and castor oil produced no stools. The vomiting ceased, but there was extreme tenderness in the right iliac region, and the patient expired on the 14th, having experienced for many days the most excruciating sufferings.

*Sectio Cadaveris.* On opening the abdomen the small intestines appeared much distended. The omentum had contracted adhesions in the right iliac fossa, and formed a flattened band, adhering towards the middle of the mesentery, and forming an arch beneath which the distended intestine passed twice. The gut, where pressed on by the band, exhibited a depression, but not of sufficient depth to prevent fæcal matters from travelling onwards. Beneath the two convolutions, the ileum, about two feet from the valve, formed two other turns, united to the neighbouring parts, and remarkably contracted opposite the band of the omentum. Had the latter been divided the strangulation would have ceased. Above this part the intestines were distended, below they were shrunken and empty. At the point of constriction the intestine was livid but not gangrenous; the peritoneum was somewhat injected and shewed recent lymph; the lungs were filled with tubercles.

*Case* 2. Pierre Foyer, ætatis 24, brass-founder, was admitted on the 20th October, 1829, having suffered for some time from the colic of his trade. For three days it had been particularly severe, with tension of the belly, nausea, and constipation; the pulse was quiet, the tongue natural. Some croton oil was given him, but next day the belly was rather more tense and painful, the pulse more frequent; he had vomited twice, and had not had a stool for seven days. Leeches, lavements, warm-baths, and demulcents, were the means resorted to without relief. The pain on the 22d was very severe and increased by pressure; there was tension in the left iliac region and depression in the right, and the fever was augmented. Bleeding and leeches were employed with no benefit. The umbilicus and especially the left iliac region became prominent and tense, the patient could only recline on the right side or with the body bent forwards, and the aspect betrayed extreme anxiety. Bleeding procured a temporary respite from pain, and so did leeches, but he sank at one o'clock next morning.

* Journ. Hebdomad. No. 95.

*Sectio Cadaveris.* The small intestines were much inflated with air, and united together by soft lymph. The stomach was dragged to the left, and so were the cæcum and ascending colon, which was placed beside the descending, throughout the whole of its length; the right half of the transverse portion was consequently doubled on the left, which had been the cause of the displacement of the stomach. From the right edge of the liver, behind the gall bladder, a band passed across the intestines, dividing the abdomen into a superior and inferior portion, and directing its course to the middle of the left iliac fossa. Here the intestine was enormously distended and formed a sort of tumour round which the band gave two turns, and finally terminated by continuity with the mesentery. Out of the noose thus formed the intestine could not be withdrawn, in consequence of its distention, but when evacuated of upwards of two pints of fluid and air, the reduction was effected. The intestine implicated was the ileum, part of the ileo-cœcal valve, and the ascending part of the colon towards its transverse arch. The gut being withdrawn, the band was then much longer than the breadth of the abdominal cavity. The intestines were a little reddened, the strangulated portion was red, ecchymosed, rather livid in some parts, but not affected with gangrene. The omentum was deficient in several points.

The reporter of these cases, M. Bonnet, a clinique interne, labours hard to prove that an operation was not only proper, but practicable, and likely to be successful in the preceding cases. He thinks that the diagnosis of an internal strangulation is easy; we believe that it is occasionally not difficult. But the question, *where* that strangulation is, demands a little more consideration; for it is not to be tolerated that we should cut into a patient's belly, to look for bridles and bands of lymph, which possibly may not exist, or, if present, may be inaccessible to the surgeon's knife. M. Bonnet, indeed, affirms that in the preceding cases it was neither difficult to pronounce on internal strangulation, to determine its situation, nor to have performed an operation for its relief. Of the two latter propositions we entertain very serious doubts, and although the issue precludes the decision of the point, yet we think the medical attendants did wisely in not imbruing their hands in blood. We do not affirm that the operation can never be proper, or that circumstances may not arise to point out the nature of a case, and the part producing the strangulation, in such a manner as to render the interference of the surgeon desirable. But we are quite sure that such beacons do not light us on our way in the great majority of these cases.

### II. CARBUNCLE — GANGRENE OF THE STOMACH—REMARKS.

Charles Clubot was admitted on the 28th of May, 1829, with a carbuncle on the left side of the neck. That side of the neck and the neighbouring parts were much swollen, with serum and air effused into the cellular membrane —the integuments were livid—and in the site of the carbuncle was an ulcer discharging thin yellowish fluid, produced by the patient's having scratched the part. There was a good deal of debility—the belly was slightly tender on pressure—there was some cough, with uneasiness in the left side of the chest—pains in the limbs—and extreme dejection. He had found lumps coming in his neck about a month before his admission, but the urgent symptoms only dated from the 23d. The ulcerated carbuncle was cauterized with the hot iron by M. Bonnet, and support with stimuli exhibited. On the 30th, the uneasiness in the chest was increased, and he complained of pain in the abdomen, augmented by pressure, whilst only wine was retained upon the stomach. In the night of the 31st he expired.

*Sectio Cadaveris*, 28 *hor. post mortem.* The right lung was gorged with blood. On opening the abdominal cavity, about a pint of sero-purulent fluid escaped, and recent lymph was attached to the convex surface of the stomach. The veins of the exterior of the latter, and of the omentum, were filled with blood, and the size of the stomach was nearly quadrupled. It contained little fluid when opened, but it was an inch and a half in thickness in parts, and several dark oval spots were seen on its mucous membrane. The circumference of these spots was of yellow colour, and in some of them the mucous membrane still existed, although softened, and in appearance putrid; there was no gangrenous odour. The mucous membrane was separated from the subjacent cellular tissue by a thick dark fluid. The size of the spots varied, some equalling in dimensions a six-franc piece. Two or three inches from the duodenum the jejunum presented a blackish spot, where the mucous membrane was a little softened, and a little farther on a prominence was formed by the subjacent cellular membrane being thickened and containing what was taken for blood. About the middle of the neck blood was extravasated into the cellular membrane, between the fibres of the muscles; there was a general disposition to fluidity and darkness in the blood.

Perhaps a doubt may cross the minds of our readers respecting the existence of gangrene of the stomach in this case; at all events the post mortem appearances are not satisfactory. In those who die with sloughing of the cellular membrane, and carbuncle is such, there is always a disposition to fluidity and blackness of the blood, consequently to its remora and collection in the softer tissues, and softening of the latter. These cadaveric changes take place very speedily after death, for we remember in an old man labouring under this affection, one lung was so soft as to appear quite rotten, though no more than 24 hours had elapsed between death and the examination of the body. There is also in these cases of sloughing of the cellular membrane a remarkable disposition to low and latent inflammation of the serous membranes, as the pleura or peritoneum. Thus, in the present instance there was inflammation about the peritoneal covering of the stomach.

In these cases there can be no doubt as to the proper treatment to be adopted. Wherever on pressing the integuments the subjacent cellular membrane feels boggy, or communicates a kind of emphysematous crackling, the integuments being blueish, or of dull yellow colour, with a defined and almost erysipelatous margin, there scarify. Scarify deeply, not merely down to the cellular membrane but through it, and extend the incisions beyond the limits of the affected tissue. The French use the cauterising iron, a clumsy, painful, and inefficient remedy, when put in comparison and competition with incisions. At some future time we shall take an opportunity of making some more extensive observations on sloughing of the cellular membrane, a subject to which we have paid some attention, and on which we could communicate some interesting facts.

---

